# Impact of nonspecific allograft biopsy findings in symptomatic kidney transplant recipients

**DOI:** 10.1038/s41598-024-54596-7

**Published:** 2024-02-18

**Authors:** Bon Jin Koo, Hyuk Huh, Byung Min Ye, Yunmi Kim, Byung Hyun Choi, Hyun Jeong Lee, Mi Seon Kang, Dong Won Lee, Soo Bong Lee, Yeong Hoon Kim, Il Young Kim, Taehee Kim, Seo Rin Kim

**Affiliations:** 1 Division of Nephrology, Department of Internal Medicine, Pusan National University School of Medicine, Yangsan, South Korea; 2 Research Institute for Convergence of Biomedical Science and Technology, Pusan National University Yangsan Hospital, Yangsan, South Korea; 3 Department of Internal Medicine-Nephrology, Inje University Busan Paik Hospital, Busan, South Korea; 4 Department of Surgery, Pusan National University School of Medicine, Yangsan, South Korea; 5 Department of Pathology, Pusan National University School of Medicine, Yangsan, South Korea; 6 Department of Pathology, Inje University Busan Paik Hospital, Busan, South Korea

**Keywords:** Allograft, Kidney transplantation, For-cause biopsy, Indication biopsy, Nephrology, Renal replacement therapy

## Abstract

A for-cause biopsy is performed to diagnose the cause of allograft dysfunction in kidney transplantation. We occasionally encounter ambiguous biopsy results in symptomatic kidney transplant recipients. Yet, the allograft survival outcome in symptomatic recipients with nonspecific allograft biopsy findings remains unclear. The purpose of this study was to analyze the impact of nonspecific for-cause biopsy findings in symptomatic kidney transplant recipients. We retrospectively collected records from 773 kidney transplant recipients between January 2008 and October 2021. The characteristics of transplant recipients with nonspecific findings in the first for-cause biopsy were analyzed. Nonspecific allograft biopsy findings were defined as other biopsy findings excluding rejection, borderline rejection, calcineurin inhibitor toxicity, infection, glomerulonephritis, and diabetic nephropathy. The graft outcome was compared between recipients who had never undergone a for-cause biopsy and those who had a first for-cause biopsy with nonspecific findings. The graft survival in recipients with nonspecific for-cause biopsy findings was comparable to that in recipients who did not require the for-cause biopsy before and after propensity score matching. Even in symptomatic kidney transplant recipients, nonspecific allograft biopsy findings might not be a poor prognostic factor for allograft survival compared to recipients who did not require the for-cause biopsy.

## Introduction

Allograft dysfunction is one of the most critical clinical situations for recipients of kidney transplantation. Even though a variety of predictive and prognostic markers are being used in clinical practice^1,2^, the allograft biopsy is still the most precise diagnostic tool to clarify causes of allograft dysfunction, such as acute rejection, infection, drug toxicity, or glomerulonephritis recurrence.

The allograft-for-cause biopsy is performed in cases of allograft dysfunction like estimated glomerular filtration rate decline, failure of serum creatinine decline, increased proteinuria, or lasting delayed graft function (DGF). Based on biopsy results, major abnormalities like antibody-mediated rejection (ABMR), T-cell-mediated rejection (TCMR), mixed rejection, borderline rejection, and calcineurin inhibitor (CNI) toxicity promote the adjustment of immunosuppressive agents. In addition, therapeutic consideration could be necessary for specific findings such as infection, chronic change, glomerular diseases, and other minor findings. In one cohort study, the for-cause biopsy group in the first 2 weeks after kidney transplantation had a worse outcome compared with those without an early biopsy^3^. It is well known that acute rejection after kidney transplantation negatively affects long-term renal outcomes^4,5^. And even subclinical inflammation might have a worse prognosis for the outcome of kidney transplantation^6–9^.

However, there is a lack of study about nonspecific allograft biopsy findings at for-cause biopsy, which are occasionally encountered in situations with allograft dysfunction. According to some retrospective studies, 20–29% of recipients had a renal biopsy result with minor abnormalities, such as nonspecific findings in allograft for-cause biopsy^10–12^. In this paper, we studied kidney transplant recipients with nonspecific for-cause biopsy findings and evaluated the allograft survival of these patients.

## Results

**Figure 1:**
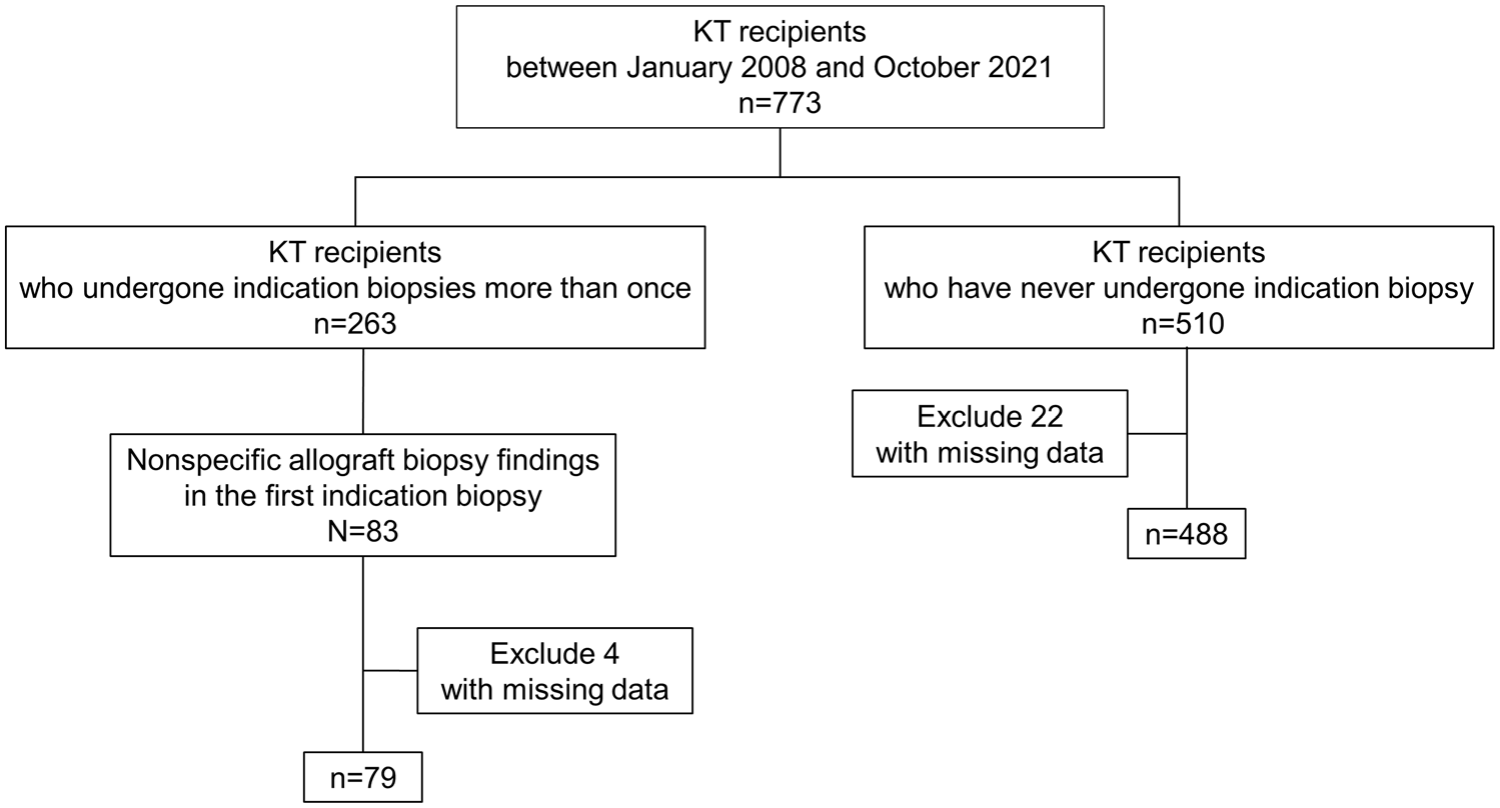
Study design and overview. KT, kidney transplantation.

A total of 263 out of 773 patients had the for-cause biopsy during the study period, whereas 510 recipients had never undergone the graft biopsy (Fig. 1). Biopsy results showed ABMR, TCMR, borderline rejection, CNI toxicity, infection, glomerulonephritis, or diabetic nephropathy in 180 out of 263 patients. Seventy-nine patients with nonspecific allograft biopsy findings, excluding four patients with missing data, were finally enrolled. We compared 79 recipients with nonspecific allograft biopsy findings and 488 recipients who had never undergone the for-cause biopsy, excluding 22 patients with missing data (Table 1). The mean age at transplant is younger in patients with nonspecific allograft biopsy findings (42.6, SD 11.7) than in patients with no for-cause biopsy (46.6, SD 11.3) (*P* = 0.004). The proportion of deceased donor kidney transplantation tended to be higher in patients with nonspecific allograft biopsy findings (54.4%) than in patients with no for-cause biopsy (43.0%) (P = 0.06). The use of antithymocyte globulin (ATG) induction therapy is less observed in patients with nonspecific allograft biopsy findings (5.4%) than in patients with no for-cause biopsy (18.8%) (*P* = 0.004). There are no statistical differences between both groups with respect to sex, duration of dialysis, ABO compatibility, or the number of human leukocyte antigen (HLA) mismatches.

**Table 1:** Patient demographics and clinical characteristics.

		Nonspecific for-cause biopsy findings N = 79	No for-cause Biopsy N = 488	P value
Age at transplant, mean (SD), y		42.6 (11.7)	46.6 (11.3)	0.004
Sex, No	Female	30 (38.0%)	233 (47.7%)	
	Male	49 (62.0%)	255 (52.3%)	0.11
Causes of ESKD^a^	DM	19.5%	21.9%	
	HTN	19.5%	12.8%	
	Chronic GN	31.2%	25.9%	
	ADPKD	3.9%	5.8%	
	Others^b^	3.9%	3.5%	
	Unknown	22.1%	30.0%	
Types of donor, No	Living	36 (45.6%)	278 (57.0%)	
	Deceased	43 (54.4%)	210 (43.0%)	0.06
Preemptive KT, No		14 (17.7%)	67 (13.7%)	0.35
Dialysis vintages, y, mean (SD)^c^		3.98 (3.10)	4.67 (4.87)	0.28
ABO-incompatible KT, No		5 (6.3%)	59 (12.1%)	0.13
HLA mismatching, mean (SD)		3.61 (1.66)	3.52 (1.67)	0.68
Induction therapy, No^d^	Basiliximab	70 (94.6%)	380 (81.2%)	
	ATG	4 (5.4%)	88 (18.8%)	0.004

Causes of ESKD ^a^Two missing data at nonspecific for-cause biopsy findings group and five missing data at no for-cause biopsy group.

Others ^b^Consist of obstructive uropathy, urinary tract infection, malignancy, nephrotoxic agents, hereditary nephropathy, etc.

Dialysis vintages, y, mean (SD) ^c^Missing value 15 (17%) and 105 (21%) at nonspecific for-cause biopsy findings and no for-cause biopsy groups.

Induction therapy, No ^d^Missing value 5 (6.3%) at nonspecific for-cause biopsy findings and 20 (4.1%) at no for-cause biopsy groups.

ADPKD, autosomal dominant polycystic kidney disease; ATG, antithymocyte globulin; DM, diabetes mellitus; ESKD, end stage kidney disease; GN, glomerulonephritis; HLA, human leukocyte antigen; HTN, hypertension; SD, standard deviation; KT, kidney transplantation.

### The causes and biopsy time point of the first for-cause biopsy in patients with nonspecific findings

**Figure 2:**
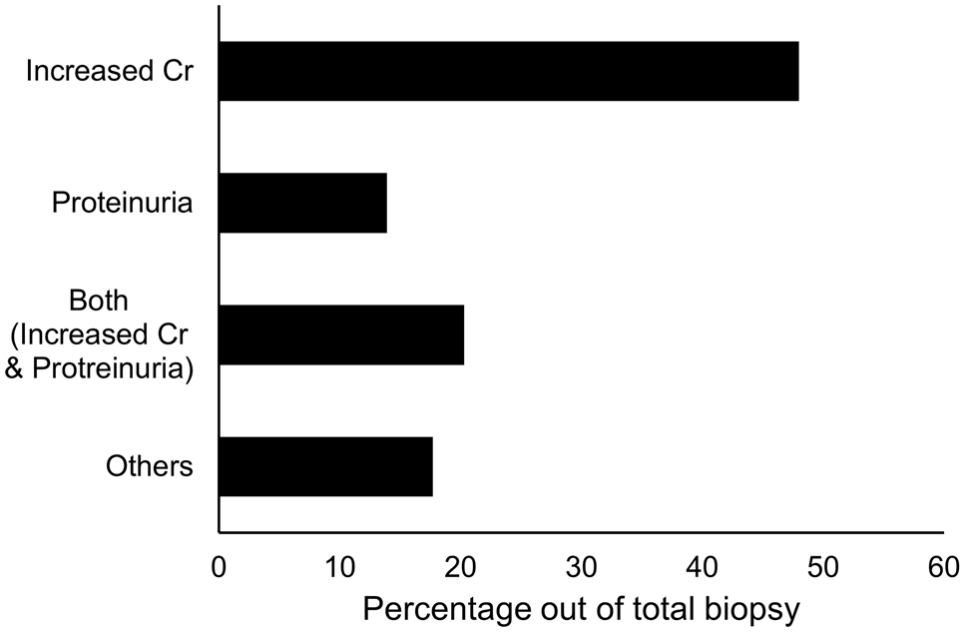
The causes of the first for-cause biopsy in patients with nonspecific allograft findings. Increased creatinine levels (48.1%), proteinuria (13.9%), both (20.3%), and others (17.7%). Others included the detection of de novo donor-specific antibodies, a high Doppler restrictive index (> 0.8), and any other decisions made by medical staff. Cr, creatinine.

**Figure 3:**
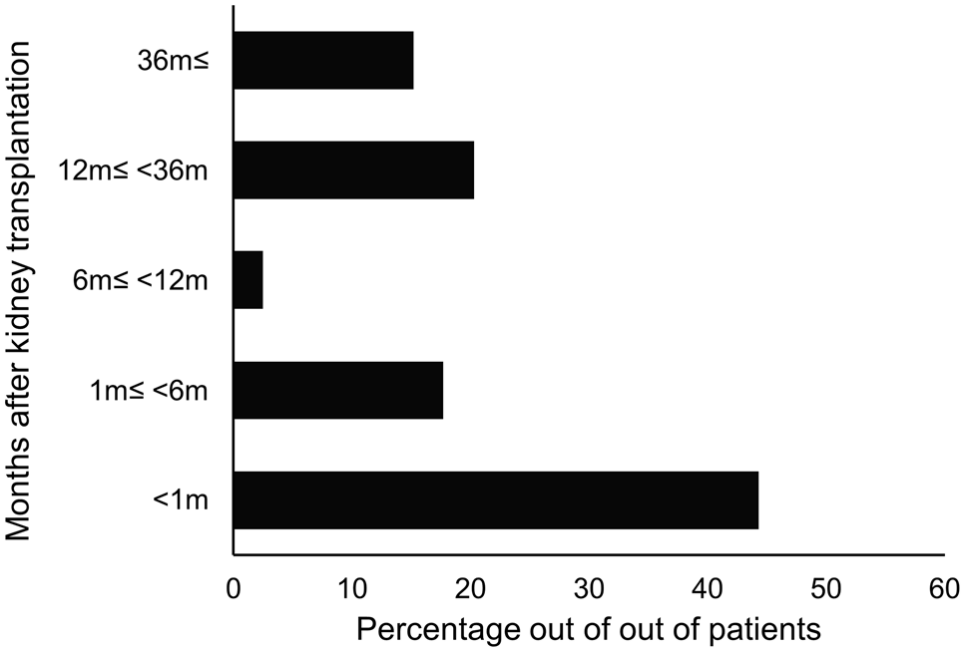
The biopsy time point in patients with nonspecific allograft findings. The distribution of biopsy time points is as follows: 44.3% of patients with nonspecific allograft findings within 1 month after kidney transplantation, 17.7% at 1–6 months, 2.5% at 6–12 months, 20.3% at 12–36 months, and 15.2% after 36 months.

The causes of the for-cause biopsy in patients with nonspecific allograft biopsy findings were increased creatinine levels (48.1%; mean creatinine changes, 0.36 ± 0.82 mg/dL), proteinuria (13.9%; mean urine protein-to-creatinine ratio (PCR), 721.7 ± 2377.8 mg/g), both (20.3%), and others (17.7%). Others included the detection of de novo donor-specific antibodies, high Doppler resistant index (RI) (> 0.8), and any other cases deemed necessary by medical staff (Fig. 2). The biopsy time point of the first for-cause biopsy after kidney transplantation in patients with nonspecific allograft biopsy findings was 15.4 ± 27.5 months. Nonspecific allograft biopsy findings were present in 44.3% of patients within 1 month after kidney transplantation, 17.7% at 1–6 months, 2.5% at 6–12 months, 20.3% at 12–36 months, and 15.2% after 36 months (Fig. 3). Interestingly, over 60% of patients with nonspecific allograft biopsy findings had a for-cause biopsy within 12 months after kidney transplantation.

### The following biopsy findings in patients with nonspecific allograft findings

Among patients with nonspecific findings in the first for-cause biopsy, 31.6% and 10.1% had the second and third allograft biopsies, respectively. In the second biopsy, 28% of recipients had nonspecific allograft findings, followed by ABMR (20%) and TCMR (20%). Following TCMR (25%) and glomerulonephritis (25%), 25% of the third biopsy had persistent nonspecific allograft findings (Table 2).

**Table 2:** The following biopsy findings after the first nonspecific for-cause biopsy result.

Biopsy findings		The second for-cause biopsy		The third for-cause biopsy	
Nonspecific		7	28%	2	25%
ABMR		5	20%	0	0%
TCMR		5	20%	2	25%
ABMR + TCMR		1	4%	0	0%
Borderline rejection		0	0%	0	0%
CNI toxicity		0	0%	0	0%
Infection		1	4%	1	12.5%
GN	Only GN	2		1	
	Combined ABMR	2	24%	1	25%
	Combined TCMR	2		0	
DM		0	0%	1	12.5%
Total		N = 25	100%	N = 8	100%

ABMR, antibody-mediated rejection; CNI, calcineurin inhibitor; DM, diabetes mellitus; GN, glomerulonephritis; TCMR, T-cell-mediated rejection.

### The graft survival in kidney transplantation recipients with nonspecific allograft findings

**Figure 4:**
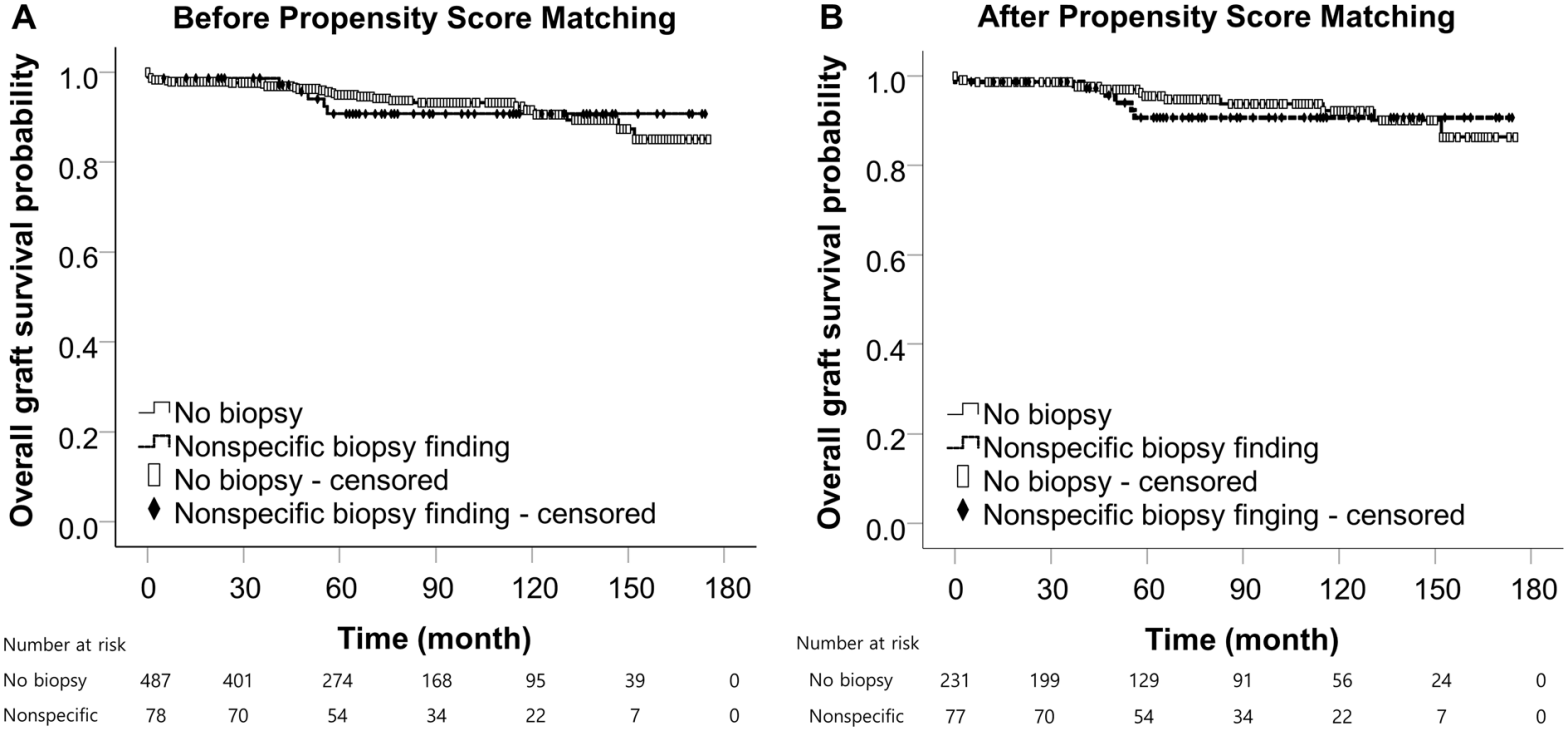
Survival analysis in patients with nonspecific allograft findings. (**A**) Graft survival by Kaplan–Meier analysis using the Log Rank test (Mantel-Cox). *P* = 0.85. (**B**) Graft survival using the Log Rank test after 1:3 propensity score matching by age at transplant and sex. *P* = 0.67

**Figure 5:**
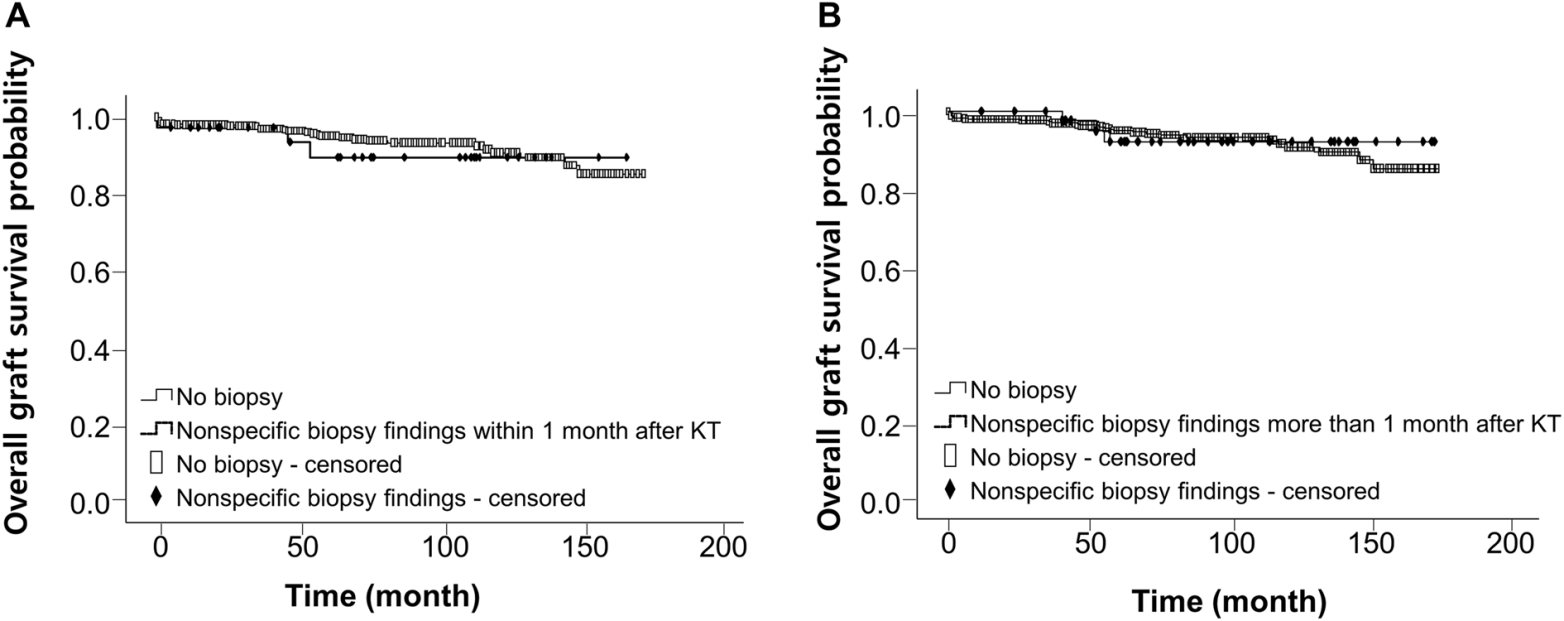
Survival analysis over the biopsy time in patients with nonspecific allograft findings. Graft survival in recipients with nonspecific for-cause biopsy findings within (**A**) and more than (**B**) one month after kidney transplantation by Kaplan–Meier analysis using the Log Rank test (Mantel-Cox). *P* = 0.60 (**A**) and *P* = 0.85 (**B**).

In Kaplan–Meier survival analysis, the graft survival in recipients with nonspecific for-cause biopsy findings was comparable to that in recipients who did not require the for-cause biopsy (*P* = 0.85). After the 1:3 propensity score matching by age at transplant and sex, there was no significant difference in graft survival between groups (*P* = 0.67) (Fig. 4). In subgroup analysis by 1-month time point of biopsy in recipients with nonspecific allograft findings, graft survival was comparable in recipients who underwent biopsies either within 1 month (n = 35) or more than 1 month after transplantation (n = 44) compared to recipients who did not have a biopsy (*P* = 0.60 and *P* = 0.85, respectively) (Fig. 5).

## Discussion

Our study showed that 31.9% (83 out of 263) of patients who underwent a for-cause biopsy had nonspecific allograft biopsy findings in kidney transplantation. The graft outcome in recipients with nonspecific biopsy findings was comparable to that in recipients who did not require the for-cause biopsy. Interestingly, 28% and 25% of patients with nonspecific findings in the first for-cause biopsy showed a nonspecific allograft biopsy finding at the second and third for-cause biopsy, respectively. Our findings offer a better understanding of the ambiguous graft biopsy results occasionally encountered in clinical practice.

In this study, nonspecific allograft biopsy findings were defined as other findings excluding ABMR, TCMR, borderline rejection, CNI toxicity, infection, glomerulonephritis, and diabetic nephropathy. They included pathologic findings like acute tubular necrosis, tubulointerstitial nephritis, or interstitial inflammation that did not satisfy rejection. These findings were present in 31.9% of patients who underwent a for-cause biopsy. Recent studies showed that 29% of the 412 for-cause biopsies had biopsy results with “no major abnormalities”^10^ and that 25% of the 1371 kidney transplantation recipients who took a for-cause graft biopsy had “minor abnormalities”, including acute tubular injuries and tubular interstitial nephritis^11^. One domestic study revealed that 20% of 410 for-cause biopsies had pathologic findings characterized by “others”, excluding major abnormalities^12^. In addition, a previous study showed that histological diagnosis of “minor abnormalities” has comparable graft survival with “normal biopsy findings”^11^. Correspondingly, our study showed that nonspecific allograft biopsy findings might not be a poor prognostic factor even in symptomatic recipients.

In our study, patients with nonspecific allograft biopsy findings were more likely to have received a kidney from a deceased donor than patients without a for-cause biopsy. DGF is a common complication that occurs after kidney transplantation from deceased donors^13^. Nonspecific allograft biopsy findings include pathologic results such as acute tubular injury and minor vessel injury, which are often seen in DGF. Therefore, the type of donor may influence the occurrence of nonspecific allograft biopsy findings in kidney transplant recipients. Additionally, the subgroup analysis by 1-month time point of biopsy implies that graft survival may be favorable in recipients with nonspecific allograft findings, regardless of DGF. We found a significant difference in the use of ATG induction therapy between patients with nonspecific allograft biopsy findings and patients with no for-cause biopsy. ATG induction therapy is commonly used in high immunologic risk patients to reduce the incidence and severity of acute rejection^14^. Recipients with nonspecific allograft biopsy findings were at a relatively lower immunologic risk; thus, their graft survival was potentially comparable to those without a for-cause biopsy, even if they needed a for-cause biopsy.

Several studies have examined the histological diagnosis of allografts at different biopsy time points after kidney transplantation. Acute tubular injury, including CNI toxicity, was the most common finding within 14 days after kidney transplantation, accounting for 40% of all biopsies, but it decreased thereafter. On the other hand, rejection was the second most common finding within 14 days, accounting for 28% of all biopsies, and it tended to increase over time^11^. In a recent study, the histological diagnosis of “no major abnormalities” accounted for 62% of all biopsies during 0–6 weeks after kidney transplantation, 39% of biopsies during 6 weeks–6 months, 22% of biopsies during 6–12 months, and 16% of biopsies after 12 months^10^. These findings, observed relatively early after transplantation, are mainly associated with ischemic injury and acute kidney injury related to drugs or various causes. In our study, the mean biopsy time point after kidney transplantation was 15.4 months. This may be due to the inclusion of biopsy findings such as acute tubular necrosis and tubulointerstitial nephritis, which are not considered major abnormalities in other studies. Interestingly, we found that more than a quarter of patients who had nonspecific findings at the previous biopsy also had nonspecific findings at the subsequent biopsy. This suggests that these nonspecific biopsy findings may not indicate future major abnormalities, such as rejection, but rather reflect minor or transient changes in the allograft.

This study has limitations due to its retrospective observational study design, which relies on the review of electronic medical records. Regrettably, a comprehensive review of every confirmed pathologic finding was not feasible. We could not reassess histologic findings, thus not reflecting the latest Banff criteria. However, we did not include borderline rejection in nonspecific allograft findings. Therefore, we could minimize the potential variability introduced by the evolving definitions of allograft rejections over time. Variations in descriptions among pathologists were inevitable. Moreover, the decision to perform an allograft biopsy in cases of acute allograft dysfunction following kidney transplantation could vary depending on the healthcare professionals involved. The maximum follow-up period for kidney transplants in our study was 13 years, with most recipients having a follow-up duration shorter than this. Therefore, our results may not reflect a long-term graft survival beyond 10 years. Finally, there remained uncertainty concerning the definition of the nonspecific allograft biopsy findings.

In conclusion, there was no significant difference in graft survival between patients with nonspecific biopsy findings and patients with no for-cause biopsy. This study can provide more information about ambiguous graft biopsy results occasionally encountered in clinical practice. A better understanding of the minor abnormality in the allograft for-cause biopsy may help improve the management of kidney transplant recipients.

## Methods

### Patient and clinical data

This study was approved by the Institutional Review Boards of Pusan National University Yangsan Hospital (05-2022-247) and Busan-Paik Hospital (2022-11-015) with the waiver of informed consent. All methods were performed in accordance with the relevant guidelines and regulations. A total of 773 kidney transplant recipients were retrospectively enrolled at two hospitals between January 2008 and October 2021. The patients were divided into two groups according to whether the for-cause biopsies were performed. The patients underwent for-cause biopsy in cases of an increase in serum creatinine of ≥ 0.3 mg/dl from baseline, proteinuria (urine PCR ≥ 500 mg/g), lasting DGF, failure of the serum creatinine to decrease following transplantation, abnormal RI results in Doppler ultrasonography, or any other case deemed necessary by medical staff.

Clinical data and biopsy findings were reviewed from electronic medical records. Collected data included age at transplant, sex, types of donors, types and duration of dialysis, ABO compatibility, number of HLA mismatches, induction therapy, and graft survival. Nonspecific allograft biopsy findings were defined as other biopsy findings excluding ABMR, TCMR, borderline rejection, CNI toxicity, infection, glomerulonephritis, and diabetic nephropathy. We analyzed the characteristics of kidney transplantation recipients with nonspecific allograft biopsy findings at the first for-cause biopsy. We reviewed the causes of the first for-cause biopsy in patients with nonspecific allograft biopsy findings. In addition, the first biopsy time point after kidney transplantation and the following for-cause biopsy results were studied in patients with nonspecific allograft biopsy findings. The graft outcome was assessed between kidney transplant recipients with nonspecific allograft biopsy findings in the first for-cause biopsy and those who had never undergone a for-cause biopsy.

### Statistical analyses

The Kolmogorov-Sminov test was performed for normality analysis. Parametric variables were expressed as the mean and standard deviation and analyzed using an independent two-sample t-test. Nonparametric variables were analyzed with the Mann–Whitney U test. Categorical variables were expressed as numbers with percentages analyzed by the Chi-squared test or Fisher’s exact test. The Kaplan–Meier survival analysis and log-rank test were utilized to compare the graft survival rates of the groups. We applied propensity score matching analysis to minimize the influence of potential confounding biases and increase comparability between the groups. The age at transplant and sex were included to calculate the propensity scores using a multi-variate logistic regression model. A 1:3 propensity score matching method was conducted using SPSS 26.0 software (SPSS Inc., Chicago, IL, USA) and R software version 3.5.3. Statistical significance was set at *P* ≤ 0.05. All statistical analyses were done using SPSS 26.0 software.

## Data Availability

The datasets used and/or analyzed during the current study are available from the corresponding author upon reasonable request.
